# Models of Student Engagement in Music Education Classroom in Higher Education

**DOI:** 10.3389/fpsyg.2021.738207

**Published:** 2021-08-13

**Authors:** Aiqin Wang

**Affiliations:** Music School, Henan University, Kaifeng, China

**Keywords:** higher education, learning, music education, models of student, student engagement

## Abstract

Higher education is undergoing a paradigm move from passive learning toward active learning. Student engagement is assumed to be a significant criterion and gauge for the quality of the student skill for higher education; however, in the literature, the term engagement remains to be vague to delineate, and it is construed in different ways. Since institutions accentuate preparing alumnae for life further than their education, student engagement has turned out to be a priority for music education, and within the last 5 years, the attention was drawn to “Students as Partners” as a response to “students as consumers” construct manipulating higher education theory. Concerning the literature review, the meaning of student engagement, determinants influencing it, and its merits are brought together. In conclusion, the implications of student engagement are presented, and new guidelines for future research are depicted.

## Introduction

Albeit the goals of music education are predominantly in the learning of music, the extensive academic objectives of learning through music are renowned in higher education as it intends to reinforce social relationships, organizing involvement in forthcoming work life, and obtaining necessary proficiency (Sutela et al., 2020). Although studies have evidenced that music education has various mental and physical benefits such as well-being, stress reduction, and self-esteem, along with social advantages (Eerola and Eerola, 2014), many shortcomings to the music education in sophisticated professional institutions remain that mostly involve teachers' distinct academic knowledge clarification and students' self-practice teaching. Indeed, motivating students for learning is challenging; therefore, it significantly confines the efficacy of music teaching in higher vocational colleges (Xinyue, 2019). So far, a few studies have pinpointed the significance of music education (Cabedo-Mas et al., 2017; Hardcastle et al., 2017; Lasauskiene and Sun, 2019), and they have indicated that in several countries, music teachers are confronting similar difficulties related to teacher training, school music education, music curricula, or overall music education.

Recently, student engagement has been at the center of attention with more emphasis being put on education that dynamically engages learners in their learning process and often associated with student fulfillment or success (Healey et al., 2016) while it can control student frustration and alienation (Fredricks et al., 2004). In the same vein, student engagement in higher education is with no exception whichcontinues to be challenging as surveys from the 1990s straight on have frequently reported high degrees of student apprehension and a lack of confidence among students, as well (Roy et al., 2012). Fostering students' engagement is a key issue in L2 students' final achievement that can also obviate the problems in music education (Mercer, 2019).

Engagement in music education is to ensure graduates are provided with supple, new, and proficient skills that assist them to be successful in a musical milieu (Minors et al., 2017). Whereas, some claimed that learners are dissatisfied with conventional teacher-centered approaches in their classes (Garrison and Akyol, 2009), others pinpointed that students are not active regarding their learning (Healey et al., 2016).

Undoubtedly, engagement is multidimensional; a predominant “meta-construct” that attempts to explain the students' achievement (Fredricks et al., 2004), and it encompasses learning that vigorously engages learners in an extensive variety of quality proficiencies that is influential for both the academic institution and the public (Pike et al., 2011; Bakker et al., 2015). Grounded on the literature review, student engagement comprises of four distinctive related scopes, namely behavioral, affective, cognitive, and social engagement (Bowden et al., 2021). In higher education, student engagement has been moved toward behavioral perspectives that studies have revealed to be associated with high-grade learning results (Krause and Coates, 2008).

In addition, these days thanks to the rapid alterations in the syllabus, the higher education staff is trying to preserve a moderate work-life balance and needs to arrange their health and well-being to diminish job-related stress on the one hand. On the other hand, students strive for their academic achievement by trying to be successful in their dream job, where unfortunately they do not often have the required power, awareness, support, and resources. So, it is maintained that students should be invited into partnership by creating effective teamwork to form engaging learning tasks, selecting appropriate course resources, and planning fascinating evaluation items with students, not for students regarding the “students as partners” approach (Mercer-Mapstone et al., 2017). So this approach is a route that takes in staff and students learning and studying cooperatively to involve and encourage students to study their subjects meticulously and concentrate on learning results (Healey et al., 2016). In this way, both learners and the teacher have equal roles which demolish power between the two and builds equity, and a situation for collaboration (Pownall, 2020). Despite the collection of studies and reviews in this field (e.g., Hennig-Thurau et al., 2001; Xerri et al., 2018) who maintained the function of student engagement in higher education, as an emerging field, in this review article, the researcher tried to clarify students- staff partnership model in music education.

## Determinants Influencing Student Engagement

In the higher education circumstances, student engagement is adapted to the communications among the time, effort, and further pertinent means capitalized on by learners and their institutions to enhance the students' performance and status of the institution (Burch et al., 2015). However, several determinants are vital for staff, students, and institutions in this territory. Undoubtedly, the ultimate agents for successful engagement are students themselves since they must devote time and effort to academic tasks and practices that are associated with viable educational upshots (Goldsmith et al., 2017). Through interaction with these activities and practice central communicative skills, the students cultivate a disposition that leads to their creativity (Cook-Sather, 2018). In addition, such interaction also involves the staff who is another significant determinant in student engagement. When staff and students are involving dynamically in partnership around teaching objectives, students acquire skills, insights, assurance, and aptitude that govern their engagement both within and beyond the classroom (Oleson, 2016). Teachers are among the other determinants in student engagement who are motivated about what they are skillfully accountable for (Russell and Slater, 2011). Sense of belonging is interconnected to student engagement that can aid higher education institutions to inflate understandings of achievement as not only interpersonal but also individual (Cook-Sather, 2018).

### Merits of Student Engagement

Student engagement is a current concern in higher education, gradually more inspected, hypothesized, and discussed with the emergent sign of its critical role in accomplishment (Kahu, 2013). Student-staff partnership can be assumed as a cooperative, reciprocal practice in which the opportunity to collaborate is provided for all participants equally to educational conceptualization, supervisory, implementation, analysis, or inquiry (Cook-Sather et al., 2014). Moreover, through this partnership, students can be qualified with skills relevant and valuable to their future profession (Mercer-Mapstone and Bovill, 2020). Werder et al. (2012) evinced a high sense of management, accountability, and motivation during the learning procedure for learners and staff involving in partnership. In some studies, a renovated awareness of self-mindfulness along with the progress of more comprehensive teaching practices is verified (Cook-Sather and Abbot, 2016). Partnership specifies an inclination of making decisions together and generates learning involvements in cooperation that go further than discussions, making use of the creativity and extending viewpoints of both learners and teachers (Matthews, 2016).

Besides its manifold benefits aforementioned, some studies have presented the efficacy of students-staff partnership that boosts motivation (Nygaard et al., 2013; Cook-Sather et al., 2014), cultivate attentiveness and sense of identity (Dickerson et al., 2016), improves learning regarding employability expertise and graduate qualities (Pauli et al., 2016).

## Implications and Future Directions

In a student-staff partnership, taking on a counselor role develops their self-confidence as well as making them attentive to the university's academic approaches (Jensen and Bennett, 2016). Integrating the student viewpoint into educational decision-making could result in more appropriate, applicable, directed and effective socially engaged curricular activities and accordingly better learning results for students (Grant, 2019) and it allowed staff to be acquainted with their students better (Curran, 2017). Teachers' role in music education is as a good presenter, facilitator, evaluator, director, supplier to the teaching, and self-assessor (Ballantyne et al., 2012). Alongside musical and academic proficiency, teachers should also have personal potentials like organizational skills to clarify and assert concepts evidently, and they should be capable of motivating others as well (Jorgensen, 2011). In music education, students as teachers and professional composers have the freedom to explore musical ideas and concepts, while experimenting on their instruments which promotes their creative thinking (Wendzich and Andrews, 2019). By empowering students to try their instruments, they begin to discriminate what sounds and musical combinations worked well-together, and in this way, they are engaged with the issue and they commenced to problem-solve (Hickey, 2012). Sharing ideas allows students to feel group possession of the compositional work, and accordingly feeling authorized and through safe collaborative situations, they can develop empathy with the music instructors as a key educational policy (Andrews, 2016). By putting students in challenging situations, the student-teachers are supposed to collaborate to be able to control challenging situations which trigger their intercultural competence to control diverse cultures and situations and determine various ways of tackling music and music education (Broske, 2020). Due to changes in society in music education and the trouble of engaging all adherents of society in music education, enlightening expansive learning could be the preliminary point, starting with inquiring accepted practice and forming new practices which reflect both cultural and spiritual subjects and basic issues in music teaching (Engeström, 2008). Regarding studies that offer such an engagement perspective, further research is required about viable pathways toward professional development music workshops to strengthen the efficacy of student/teacher/staff collaboration and they should concentrate on educational strategies that could cultivate collaboration in music education. Proposing new learning activities, extra-curricular prospects that allow students to improve their engagement as musicians should be taken into consideration.

## Author Contributions

The author confirms being the sole contributor of this work and has approved it for publication.

## Conflict of Interest

The author declares that the research was conducted in the absence of any commercial or financial relationships that could be construed as a potential conflict of interest.

## Publisher's Note

All claims expressed in this article are solely those of the authors and do not necessarily represent those of their affiliated organizations, or those of the publisher, the editors and the reviewers. Any product that may be evaluated in this article, or claim that may be made by its manufacturer, is not guaranteed or endorsed by the publisher.
